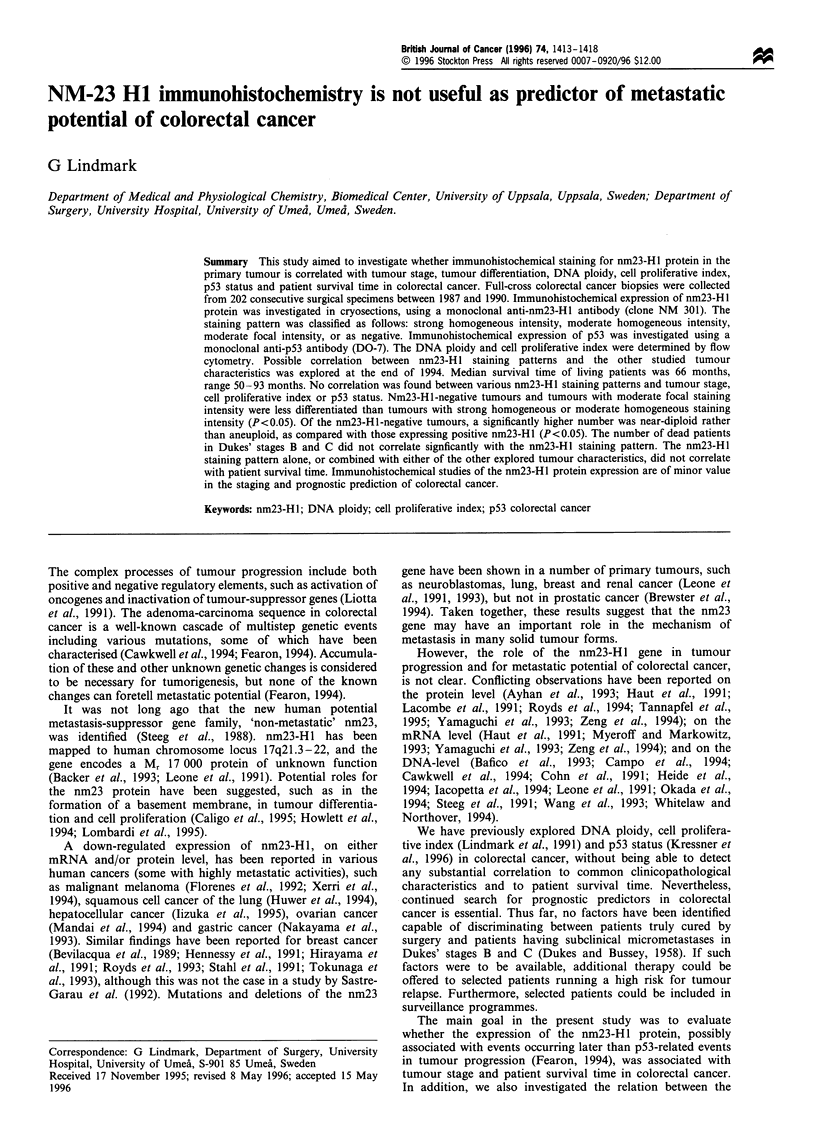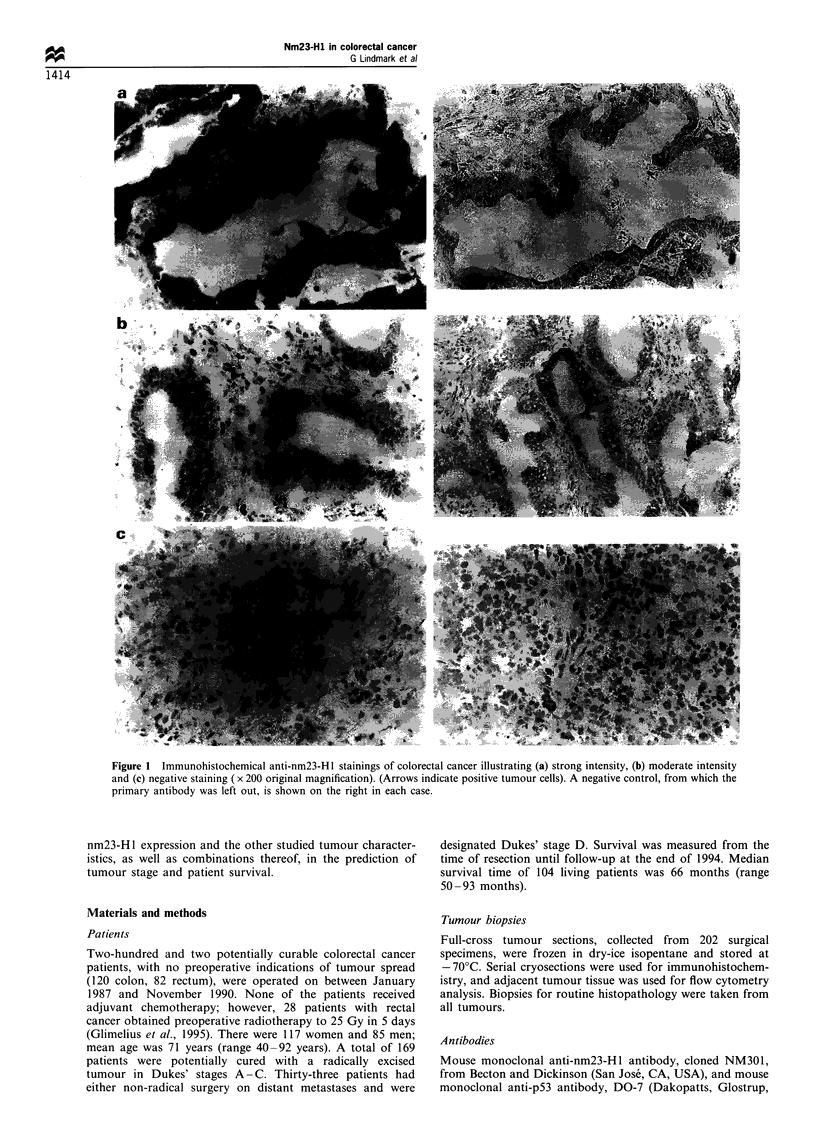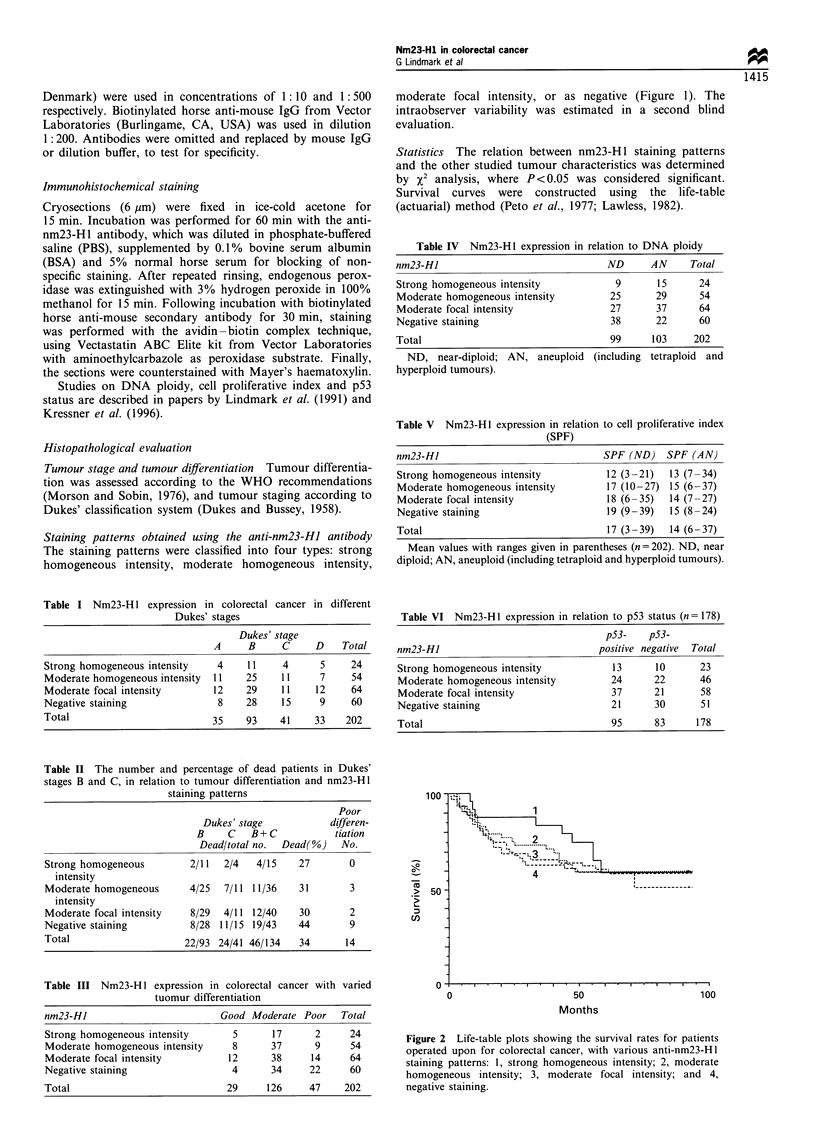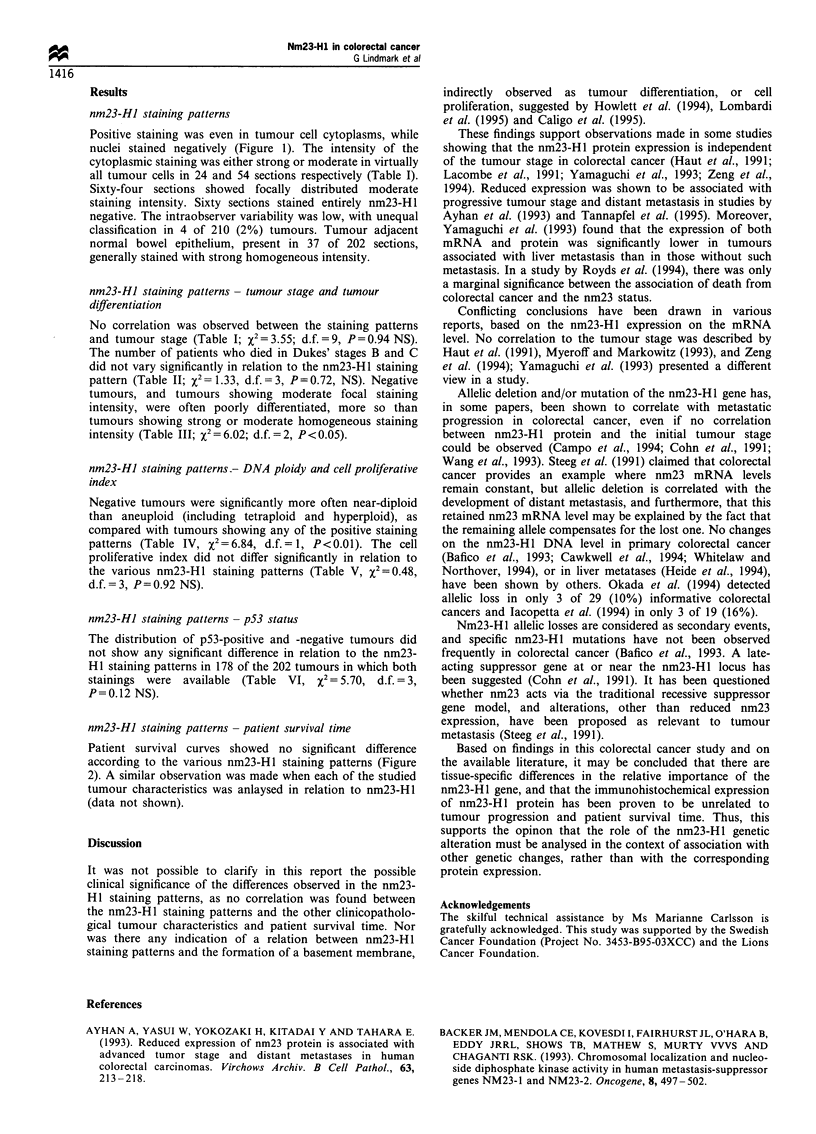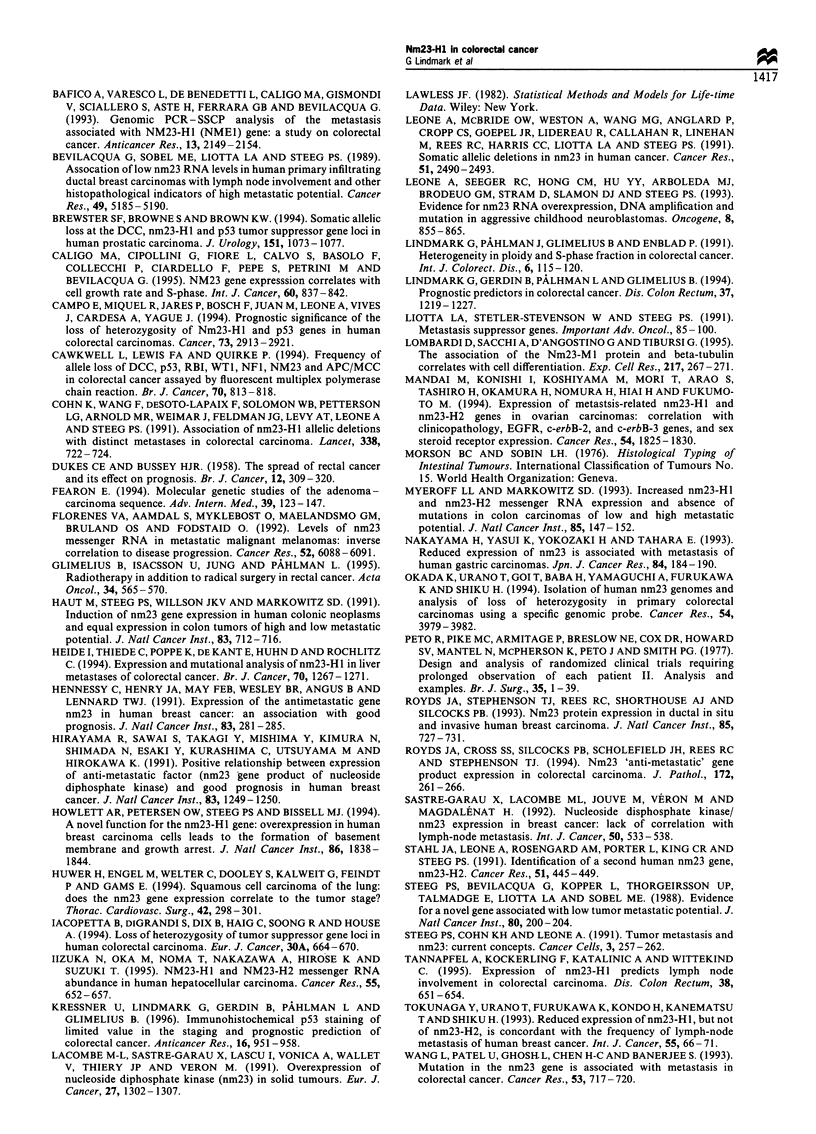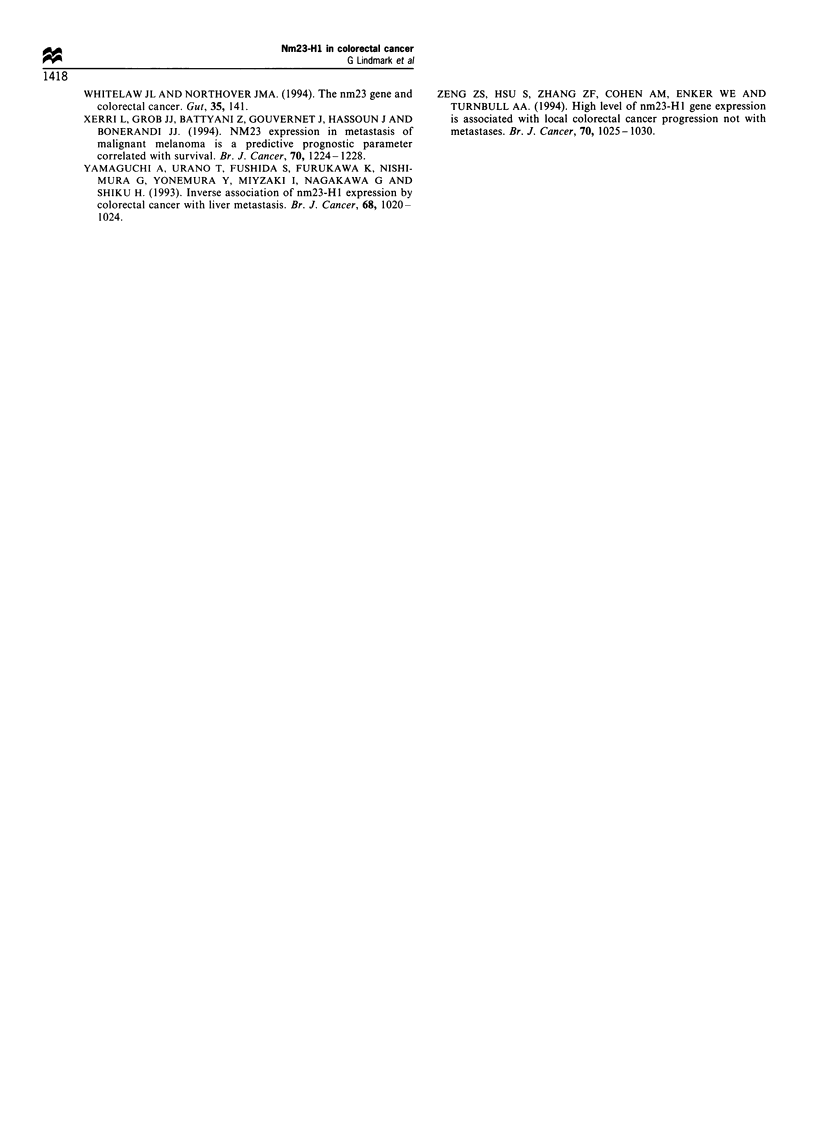# NM-23 H1 immunohistochemistry is not useful as predictor of metastatic potential of colorectal cancer.

**DOI:** 10.1038/bjc.1996.557

**Published:** 1996-11

**Authors:** G. Lindmark

**Affiliations:** Department of Medical and Physiological Chemistry, University of Uppsala, Sweden.

## Abstract

**Images:**


British Journal of Cancer (1996) 74, 1413-1418
© 1996 Stockton Press All rights reserved 0007-0920/96 $12.00

### NM-23 H1 immunohistochemistry is not useful as predictor of metastatic potential of colorectal cancer

G Lindmark

*Department of Medical and Physiological Chemistry, Biomedical Center, University of Uppsala, Uppsala, Sweden; Department of Surgery, University Hospital, University of Umeå, Umeå, Sweden.*

**Summary** This study aimed to investigate whether immunohistochemical staining for nm23-H1 protein in the primary tumour is correlated with tumour stage, tumour differentiation, DNA ploidy, cell proliferative index, p53 status and patient survival time in colorectal cancer. Full-cross colorectal cancer biopsies were collected from 202 consecutive surgical specimens between 1987 and 1990. Immunohistochemical expression of nm23-H1 protein was investigated in cryosections, using a monoclonal anti-nm23-H1 antibody (clone NM 301). The staining pattern was classified as follows: strong homogeneous intensity, moderate homogeneous intensity, moderate focal intensity, or as negative. Immunohistochemical expression of p53 was investigated using a monoclonal anti-p53 antibody (DO-7). The DNA ploidy and cell proliferative index were determined by flow cytometry. Possible correlation between nm23-H1 staining patterns and the other studied tumour characteristics was explored at the end of 1994. Median survival time of living patients was 66 months, range 50–93 months. No correlation was found between various nm23-H1 staining patterns and tumour stage, cell proliferative index or p53 status. Nm23-H1-negative tumours and tumours with moderate focal staining intensity were less differentiated than tumours with strong homogeneous or moderate homogeneous staining intensity ($P<0.05$). Of the nm23-H1-negative tumours, a significantly higher number was near-diploid rather than aneuploid, as compared with those expressing positive nm23-H1 ($P<0.05$). The number of dead patients in Dukes' stages B and C did not correlate signficantly with the nm23-H1 staining pattern. The nm23-H1 staining pattern alone, or combined with either of the other explored tumour characteristics, did not correlate with patient survival time. Immunohistochemical studies of the nm23-H1 protein expression are of minor value in the staging and prognostic prediction of colorectal cancer.

**Keywords:** nm23-H1; DNA ploidy; cell proliferative index; p53 colorectal cancer

The complex processes of tumour progression include both positive and negative regulatory elements, such as activation of oncogenes and inactivation of tumour-suppressor genes (Liotta *et al.*, 1991). The adenoma-carcinoma sequence in colorectal cancer is a well-known cascade of multistep genetic events including various mutations, some of which have been characterised (Cawkwell *et al.*, 1994; Fearon, 1994). Accumulation of these and other unknown genetic changes is considered to be necessary for tumorigenesis, but none of the known changes can foretell metastatic potential (Fearon, 1994).

It was not long ago that the new human potential metastasis-suppressor gene family, 'non-metastatic' nm23, was identified (Steeg *et al.*, 1988). nm23-H1 has been mapped to human chromosome locus 17q21.3–22, and the gene encodes a $M_r$ 17 000 protein of unknown function (Backer *et al.*, 1993; Leone *et al.*, 1991). Potential roles for the nm23 protein have been suggested, such as in the formation of a basement membrane, in tumour differentiation and cell proliferation (Caligo *et al.*, 1995; Howlett *et al.*, 1994; Lombardi *et al.*, 1995).

A down-regulated expression of nm23-H1, on either mRNA and/or protein level, has been reported in various human cancers (some with highly metastatic activities), such as malignant melanoma (Florenes *et al.*, 1992; Xerri *et al.*, 1994), squamous cell cancer of the lung (Huwer *et al.*, 1994), hepatocellular cancer (Iizuka *et al.*, 1995), ovarian cancer (Mandai *et al.*, 1994) and gastric cancer (Nakayama *et al.*, 1993). Similar findings have been reported for breast cancer (Bevilacqua *et al.*, 1989; Hennessy *et al.*, 1991; Hirayama *et al.*, 1991; Royds *et al.*, 1993; Stahl *et al.*, 1991; Tokunaga *et al.*, 1993), although this was not the case in a study by Sastre-Garau *et al.* (1992). Mutations and deletions of the nm23 gene have been shown in a number of primary tumours, such as neuroblastomas, lung, breast and renal cancer (Leone *et al.*, 1991, 1993), but not in prostatic cancer (Brewster *et al.*, 1994). Taken together, these results suggest that the nm23 gene may have an important role in the mechanism of metastasis in many solid tumour forms.

However, the role of the nm23-H1 gene in tumour progression and for metastatic potential of colorectal cancer, is not clear. Conflicting observations have been reported on the protein level (Ayhan *et al.*, 1993; Haut *et al.*, 1991; Lacombe *et al.*, 1991; Royds *et al.*, 1994; Tannapfel *et al.*, 1995; Yamaguchi *et al.*, 1993; Zeng *et al.*, 1994); on the mRNA level (Haut *et al.*, 1991; Myeroff and Markowitz, 1993; Yamaguchi *et al.*, 1993; Zeng *et al.*, 1994); and on the DNA-level (Bafico *et al.*, 1993; Campo *et al.*, 1994; Cawkwell *et al.*, 1994; Cohn *et al.*, 1991; Heide *et al.*, 1994; Iacopetta *et al.*, 1994; Leone *et al.*, 1991; Okada *et al.*, 1994; Steeg *et al.*, 1991; Wang *et al.*, 1993; Whitelaw and Northover, 1994).

We have previously explored DNA ploidy, cell proliferative index (Lindmark *et al.*, 1991) and p53 status (Kressner *et al.*, 1996) in colorectal cancer, without being able to detect any substantial correlation to common clinicopathological characteristics and to patient survival time. Nevertheless, continued search for prognostic predictors in colorectal cancer is essential. Thus far, no factors have been identified capable of discriminating between patients truly cured by surgery and patients having subclinical micrometastases in Dukes' stages B and C (Dukes and Bussey, 1958). If such factors were to be available, additional therapy could be offered to selected patients running a high risk for tumour relapse. Furthermore, selected patients could be included in surveillance programmes.

The main goal in the present study was to evaluate whether the expression of the nm23-H1 protein, possibly associated with events occurring later than p53-related events in tumour progression (Fearon, 1994), was associated with tumour stage and patient survival time in colorectal cancer. In addition, we also investigated the relation between the

Correspondence: G Lindmark, Department of Surgery, University Hospital, University of Umeå, S-901 85 Umeå, Sweden
Received 17 November 1995; revised 8 May 1996; accepted 15 May 1996

**Figure 1** Immunohistochemical anti-nm23-H1 stainings of colorectal cancer illustrating (**a**) strong intensity, (**b**) moderate intensity and (**c**) negative staining ( × 200 original magnification). (Arrows indicate positive tumour cells). A negative control, from which the primary antibody was left out, is shown on the right in each case.

nm23-H1 expression and the other studied tumour characteristics, as well as combinations thereof, in the prediction of tumour stage and patient survival.

### Materials and methods

### *Patients*

Two-hundred and two potentially curable colorectal cancer patients, with no preoperative indications of tumour spread (120 colon, 82 rectum), were operated on between January 1987 and November 1990. None of the patients received adjuvant chemotherapy; however, 28 patients with rectal cancer obtained preoperative radiotherapy to 25 Gy in 5 days (Glimelius *et al.*, 1995). There were 117 women and 85 men; mean age was 71 years (range 40–92 years). A total of 169 patients were potentially cured with a radically excised tumour in Dukes' stages A–C. Thirty-three patients had either non-radical surgery on distant metastases and were designated Dukes' stage D. Survival was measured from the time of resection until follow-up at the end of 1994. Median survival time of 104 living patients was 66 months (range 50–93 months).

### *Tumour biopsies*

Full-cross tumour sections, collected from 202 surgical specimens, were frozen in dry-ice isopentane and stored at −70°C. Serial cryosections were used for immunohistochemistry, and adjacent tumour tissue was used for flow cytometry analysis. Biopsies for routine histopathology were taken from all tumours.

### *Antibodies*

Mouse monoclonal anti-nm23-H1 antibody, cloned NM301, from Becton and Dickinson (San José, CA, USA), and mouse monoclonal anti-p53 antibody, DO-7 (Dakopatts, Glostrup,

Denmark) were used in concentrations of 1:10 and 1:500 respectively. Biotinylated horse anti-mouse IgG from Vector Laboratories (Burlingame, CA, USA) was used in dilution 1:200. Antibodies were omitted and replaced by mouse IgG or dilution buffer, to test for specificity.

*Immunohistochemical staining*

Cryosections (6 $\mu$m) were fixed in ice-cold acetone for 15 min. Incubation was performed for 60 min with the anti-nm23-H1 antibody, which was diluted in phosphate-buffered saline (PBS), supplemented by 0.1% bovine serum albumin (BSA) and 5% normal horse serum for blocking of non-specific staining. After repeated rinsing, endogenous peroxidase was extinguished with 3% hydrogen peroxide in 100% methanol for 15 min. Following incubation with biotinylated horse anti-mouse secondary antibody for 30 min, staining was performed with the avidin–biotin complex technique, using Vectastatin ABC Elite kit from Vector Laboratories with aminoethylcarbazole as peroxidase substrate. Finally, the sections were counterstained with Mayer's haematoxylin.

Studies on DNA ploidy, cell proliferative index and p53 status are described in papers by Lindmark *et al.* (1991) and Kressner *et al.* (1996).

*Histopathological evaluation*

*Tumour stage and tumour differentiation* Tumour differentiation was assessed according to the WHO recommendations (Morson and Sobin, 1976), and tumour staging according to Dukes' classification system (Dukes and Bussey, 1958).

*Staining patterns obtained using the anti-nm23-H1 antibody* The staining patterns were classified into four types: strong homogeneous intensity, moderate homogeneous intensity, moderate focal intensity, or as negative (Figure 1). The intraobserver variability was estimated in a second blind evaluation.

*Statistics* The relation between nm23-H1 staining patterns and the other studied tumour characteristics was determined by $\chi^2$ analysis, where $P<0.05$ was considered significant. Survival curves were constructed using the life-table (actuarial) method (Peto *et al.*, 1977; Lawless, 1982).

**Table I** Nm23-H1 expression in colorectal cancer in different Dukes' stages

| | Dukes' stage A | B | C | D | Total |
|---|---|---|---|---|---|
| Strong homogeneous intensity | 4 | 11 | 4 | 5 | 24 |
| Moderate homogeneous intensity | 11 | 25 | 11 | 7 | 54 |
| Moderate focal intensity | 12 | 29 | 11 | 12 | 64 |
| Negative staining | 8 | 28 | 15 | 9 | 60 |
| Total | 35 | 93 | 41 | 33 | 202 |

**Table II** The number and percentage of dead patients in Dukes' stages B and C, in relation to tumour differentiation and nm23-H1 staining patterns

| | Dukes' stage B | C | B+C | | Poor differentiation |
|---|---|---|---|---|---|
| | Dead/total no. | | | Dead(%) | No. |
| Strong homogeneous intensity | 2/11 | 2/4 | 4/15 | 27 | 0 |
| Moderate homogeneous intensity | 4/25 | 7/11 | 11/36 | 31 | 3 |
| Moderate focal intensity | 8/29 | 4/11 | 12/40 | 30 | 2 |
| Negative staining | 8/28 | 11/15 | 19/43 | 44 | 9 |
| Total | 22/93 | 24/41 | 46/134 | 34 | 14 |

**Table III** Nm23-H1 expression in colorectal cancer with varied tuomur differentiation

| *nm23-H1* | Good | Moderate | Poor | Total |
|---|---|---|---|---|
| Strong homogeneous intensity | 5 | 17 | 2 | 24 |
| Moderate homogeneous intensity | 8 | 37 | 9 | 54 |
| Moderate focal intensity | 12 | 38 | 14 | 64 |
| Negative staining | 4 | 34 | 22 | 60 |
| Total | 29 | 126 | 47 | 202 |

**Table IV** Nm23-H1 expression in relation to DNA ploidy

| *nm23-H1* | *ND* | *AN* | *Total* |
|---|---|---|---|
| Strong homogeneous intensity | 9 | 15 | 24 |
| Moderate homogeneous intensity | 25 | 29 | 54 |
| Moderate focal intensity | 27 | 37 | 64 |
| Negative staining | 38 | 22 | 60 |
| Total | 99 | 103 | 202 |

ND, near-diploid; AN, aneuploid (including tetraploid and hyperploid tumours).

**Table V** Nm23-H1 expression in relation to cell proliferative index (SPF)

| *nm23-H1* | *SPF (ND)* | *SPF (AN)* |
|---|---|---|
| Strong homogeneous intensity | 12 (3–21) | 13 (7–34) |
| Moderate homogeneous intensity | 17 (10–27) | 15 (6–37) |
| Moderate focal intensity | 18 (6–35) | 14 (7–27) |
| Negative staining | 19 (9–39) | 15 (8–24) |
| Total | 17 (3–39) | 14 (6–37) |

Mean values with ranges given in parentheses ($n=202$). ND, near diploid; AN, aneuploid (including tetraploid and hyperploid tumours).

**Table VI** Nm23-H1 expression in relation to p53 status ($n=178$)

| *nm23-H1* | *p53-positive* | *p53-negative* | *Total* |
|---|---|---|---|
| Strong homogeneous intensity | 13 | 10 | 23 |
| Moderate homogeneous intensity | 24 | 22 | 46 |
| Moderate focal intensity | 37 | 21 | 58 |
| Negative staining | 21 | 30 | 51 |
| Total | 95 | 83 | 178 |

**Figure 2** Life-table plots showing the survival rates for patients operated upon for colorectal cancer, with various anti-nm23-H1 staining patterns: 1, strong homogeneous intensity; 2, moderate homogeneous intensity; 3, moderate focal intensity; and 4, negative staining.

### Results

### nm23-H1 staining patterns

Positive staining was even in tumour cell cytoplasms, while nuclei stained negatively (Figure 1). The intensity of the cytoplasmic staining was either strong or moderate in virtually all tumour cells in 24 and 54 sections respectively (Table I). Sixty-four sections showed focally distributed moderate staining intensity. Sixty sections stained entirely nm23-H1 negative. The intraobserver variability was low, with unequal classification in 4 of 210 (2%) tumours. Tumour adjacent normal bowel epithelium, present in 37 of 202 sections, generally stained with strong homogeneous intensity.

### nm23-H1 staining patterns – tumour stage and tumour differentiation

No correlation was observed between the staining patterns and tumour stage (Table I; $\chi^2 = 3.55$; d.f. = 9, $P = 0.94$ NS). The number of patients who died in Dukes' stages B and C did not vary significantly in relation to the nm23-H1 staining pattern (Table II; $\chi^2 = 1.33$, d.f. = 3, $P = 0.72$, NS). Negative tumours, and tumours showing moderate focal staining intensity, were often poorly differentiated, more so than tumours showing strong or moderate homogeneous staining intensity (Table III; $\chi^2 = 6.02$; d.f. = 2, $P < 0.05$).

### nm23-H1 staining patterns – DNA ploidy and cell proliferative index

Negative tumours were significantly more often near-diploid than aneuploid (including tetraploid and hyperploid), as compared with tumours showing any of the positive staining patterns (Table IV, $\chi^2 = 6.84$, d.f. = 1, $P < 0.01$). The cell proliferative index did not differ significantly in relation to the various nm23-H1 staining patterns (Table V, $\chi^2 = 0.48$, d.f. = 3, $P = 0.92$ NS).

### nm23-H1 staining patterns – p53 status

The distribution of p53-positive and -negative tumours did not show any significant difference in relation to the nm23-H1 staining patterns in 178 of the 202 tumours in which both stainings were available (Table VI, $\chi^2 = 5.70$, d.f. = 3, $P = 0.12$ NS).

### nm23-H1 staining patterns – patient survival time

Patient survival curves showed no significant difference according to the various nm23-H1 staining patterns (Figure 2). A similar observation was made when each of the studied tumour characteristics was anlaysed in relation to nm23-H1 (data not shown).

### Discussion

It was not possible to clarify in this report the possible clinical significance of the differences observed in the nm23-H1 staining patterns, as no correlation was found between the nm23-H1 staining patterns and the other clinicopathological tumour characteristics and patient survival time. Nor was there any indication of a relation between nm23-H1 staining patterns and the formation of a basement membrane, indirectly observed as tumour differentiation, or cell proliferation, suggested by Howlett *et al.* (1994), Lombardi *et al.* (1995) and Caligo *et al.* (1995).

These findings support observations made in some studies showing that the nm23-H1 protein expression is independent of the tumour stage in colorectal cancer (Haut *et al.*, 1991; Lacombe *et al.*, 1991; Yamaguchi *et al.*, 1993; Zeng *et al.*, 1994). Reduced expression was shown to be associated with progressive tumour stage and distant metastasis in studies by Ayhan *et al.* (1993) and Tannapfel *et al.* (1995). Moreover, Yamaguchi *et al.* (1993) found that the expression of both mRNA and protein was significantly lower in tumours associated with liver metastasis than in those without such metastasis. In a study by Royds *et al.* (1994), there was only a marginal significance between the association of death from colorectal cancer and the nm23 status.

Conflicting conclusions have been drawn in various reports, based on the nm23-H1 expression on the mRNA level. No correlation to the tumour stage was described by Haut *et al.* (1991), Myeroff and Markowitz (1993), and Zeng *et al.* (1994); Yamaguchi *et al.* (1993) presented a different view in a study.

Allelic deletion and/or mutation of the nm23-H1 gene has, in some papers, been shown to correlate with metastatic progression in colorectal cancer, even if no correlation between nm23-H1 protein and the initial tumour stage could be observed (Campo *et al.*, 1994; Cohn *et al.*, 1991; Wang *et al.*, 1993). Steeg *et al.* (1991) claimed that colorectal cancer provides an example where nm23 mRNA levels remain constant, but allelic deletion is correlated with the development of distant metastasis, and furthermore, that this retained nm23 mRNA level may be explained by the fact that the remaining allele compensates for the lost one. No changes on the nm23-H1 DNA level in primary colorectal cancer (Bafico *et al.*, 1993; Cawkwell *et al.*, 1994; Whitelaw and Northover, 1994), or in liver metatases (Heide *et al.*, 1994), have been shown by others. Okada *et al.* (1994) detected allelic loss in only 3 of 29 (10%) informative colorectal cancers and Iacopetta *et al.* (1994) in only 3 of 19 (16%).

Nm23-H1 allelic losses are considered as secondary events, and specific nm23-H1 mutations have not been observed frequently in colorectal cancer (Bafico *et al.*, 1993. A late-acting suppressor gene at or near the nm23-H1 locus has been suggested (Cohn *et al.*, 1991). It has been questioned whether nm23 acts via the traditional recessive suppressor gene model, and alterations, other than reduced nm23 expression, have been proposed as relevant to tumour metastasis (Steeg *et al.*, 1991).

Based on findings in this colorectal cancer study and on the available literature, it may be concluded that there are tissue-specific differences in the relative importance of the nm23-H1 gene, and that the immunohistochemical expression of nm23-H1 protein has been proven to be unrelated to tumour progression and patient survival time. Thus, this supports the opinon that the role of the nm23-H1 genetic alteration must be analysed in the context of association with other genetic changes, rather than with the corresponding protein expression.

**Acknowledgements**
The skilful technical assistance by Ms Marianne Carlsson is gratefully acknowledged. This study was supported by the Swedish Cancer Foundation (Project No. 3453-B95-03XCC) and the Lions Cancer Foundation.

### References

AYHAN A, YASUI W, YOKOZAKI H, KITADAI Y AND TAHARA E. (1993). Reduced expression of nm23 protein is associated with advanced tumor stage and distant metastases in human colorectal carcinomas. *Virchows Archiv. B Cell Pathol.*, **63**, 213–218.

BACKER JM, MENDOLA CE, KOVESDI I, FAIRHURST JL, O'HARA B, EDDY JRRL, SHOWS TB, MATHEW S, MURTY VVVS AND CHAGANTI RSK. (1993). Chromosomal localization and nucleoside diphosphate kinase activity in human metastasis-suppressor genes NM23-1 and NM23-2. *Oncogene*, **8**, 497–502.

BAFICO A, VARESCO L, DE BENEDETTI L, CALIGO MA, GISMONDI V, SCIALLERO S, ASTE H, FERRARA GB AND BEVILACQUA G. (1993). Genomic PCR-SSCP analysis of the metastasis associated with NM23-H1 (NME1) gene: a study on colorectal cancer. *Anticancer Res.*, **13**, 2149-2154.

BEVILACQUA G, SOBEL ME, LIOTTA LA AND STEEG PS. (1989). Assocation of low nm23 RNA levels in human primary infiltrating ductal breast carcinomas with lymph node involvement and other histopathological indicators of high metastatic potential. *Cancer Res.*, **49**, 5185-5190.

BREWSTER SF, BROWNE S AND BROWN KW. (1994). Somatic allelic loss at the DCC, nm23-H1 and p53 tumor suppressor gene loci in human prostatic carcinoma. *J. Urology*, **151**, 1073-1077.

CALIGO MA, CIPOLLINI G, FIORE L, CALVO S, BASOLO F, COLLECCHI P, CIARDELLO F, PEPE S, PETRINI M AND BEVILACQUA G. (1995). NM23 gene expresssion correlates with cell growth rate and S-phase. *Int. J. Cancer*, **60**, 837-842.

CAMPO E, MIQUEL R, JARES P, BOSCH F, JUAN M, LEONE A, VIVES J, CARDESA A, YAGUE J. (1994). Prognostic significance of the loss of heterozygosity of Nm23-H1 and p53 genes in human colorectal carcinomas. *Cancer*, **73**, 2913-2921.

CAWKWELL L, LEWIS FA AND QUIRKE P. (1994). Frequency of allele loss of DCC, p53, RBI, WT1, NF1, NM23 and APC/MCC in colorectal cancer assayed by fluorescent multiplex polymerase chain reaction. *Br. J. Cancer*, **70**, 813-818.

COHN K, WANG F, DESOTO-LAPAIX F, SOLOMON WB, PETTERSON LG, ARNOLD MR, WEIMAR J, FELDMAN JG, LEVY AT, LEONE A AND STEEG PS. (1991). Association of nm23-H1 allelic deletions with distinct metastases in colorectal carcinoma. *Lancet*, **338**, 722-724.

DUKES CE AND BUSSEY HJR. (1958). The spread of rectal cancer and its effect on prognosis. *Br. J. Cancer*, **12**, 309-320.

FEARON E. (1994). Molecular genetic studies of the adenoma-carcinoma sequence. *Adv. Intern. Med.*, **39**, 123-147.

FLORENES VA, AAMDAL S, MYKLEBOST O, MAELANDSMO GM, BRULAND OS AND FODSTAID O. (1992). Levels of nm23 messenger RNA in metastatic malignant melanomas: inverse correlation to disease progression. *Cancer Res.*, **52**, 6088-6091.

GLIMELIUS B, ISACSSON U, JUNG AND PÅHLMAN L. (1995). Radiotherapy in addition to radical surgery in rectal cancer. *Acta Oncol.*, **34**, 565-570.

HAUT M, STEEG PS, WILLSON JKV AND MARKOWITZ SD. (1991). Induction of nm23 gene expression in human colonic neoplasms and equal expression in colon tumors of high and low metastatic potential. *J. Natl Cancer Inst.*, **83**, 712-716.

HEIDE I, THIEDE C, POPPE K, DE KANT E, HUHN D AND ROCHLITZ C. (1994). Expression and mutational analysis of nm23-H1 in liver metastases of colorectal cancer. *Br. J. Cancer*, **70**, 1267-1271.

HENNESSY C, HENRY JA, MAY FEB, WESLEY BR, ANGUS B AND LENNARD TWJ. (1991). Expression of the antimetastatic gene nm23 in human breast cancer: an association with good prognosis. *J. Natl Cancer Inst.*, **83**, 281-285.

HIRAYAMA R, SAWAI S, TAKAGI Y, MISHIMA Y, KIMURA N, SHIMADA N, ESAKI Y, KURASHIMA C, UTSUYAMA M AND HIROKAWA K. (1991). Positive relationship between expression of anti-metastatic factor (nm23 gene product of nucleoside diphosphate kinase) and good prognosis in human breast cancer. *J. Natl Cancer Inst.*, **83**, 1249-1250.

HOWLETT AR, PETERSEN OW, STEEG PS AND BISSELL MJ. (1994). A novel function for the nm23-H1 gene: overexpression in human breast carcinoma cells leads to the formation of basement membrane and growth arrest. *J. Natl Cancer Inst.*, **86**, 1838-1844.

HUWER H, ENGEL M, WELTER C, DOOLEY S, KALWEIT G, FEINDT P AND GAMS E. (1994). Squamous cell carcinoma of the lung: does the nm23 gene expression correlate to the tumor stage? *Thorac. Cardiovasc. Surg.*, **42**, 298-301.

IACOPETTA B, DIGRANDI S, DIX B, HAIG C, SOONG R AND HOUSE A. (1994). Loss of heterozygosity of tumor suppressor gene loci in human colorectal carcinoma. *Eur. J. Cancer*, **30A**, 664-670.

IIZUKA N, OKA M, NOMA T, NAKAZAWA A, HIROSE K AND SUZUKI T. (1995). NM23-H1 and NM23-H2 messenger RNA abundance in human hepatocellular carcinoma. *Cancer Res.*, **55**, 652-657.

KRESSNER U, LINDMARK G, GERDIN B, PÅHLMAN L AND GLIMELIUS B. (1996). Immunohistochemical p53 staining of limited value in the staging and prognostic prediction of colorectal cancer. *Anticancer Res.*, **16**, 951-958.

LACOMBE M-L, SASTRE-GARAU X, LASCU I, VONICA A, WALLET V, THIERY JP AND VERON M. (1991). Overexpression of nucleoside diphosphate kinase (nm23) in solid tumours. *Eur. J. Cancer*, **27**, 1302-1307.

LAWLESS JF. (1982). *Statistical Methods and Models for Life-time Data*. Wiley: New York.

LEONE A, MCBRIDE OW, WESTON A, WANG MG, ANGLARD P, CROPP CS, GOEPEL JR, LIDEREAU R, CALLAHAN R, LINEHAN M, REES RC, HARRIS CC, LIOTTA LA AND STEEG PS. (1991). Somatic allelic deletions in nm23 in human cancer. *Cancer Res.*, **51**, 2490-2493.

LEONE A, SEEGER RC, HONG CM, HU YY, ARBOLEDA MJ, BRODEUO GM, STRAM D, SLAMON DJ AND STEEG PS. (1993). Evidence for nm23 RNA overexpression, DNA amplification and mutation in aggressive childhood neuroblastomas. *Oncogene*, **8**, 855-865.

LINDMARK G, PÅHLMAN J, GLIMELIUS B AND ENBLAD P. (1991). Heterogeneity in ploidy and S-phase fraction in colorectal cancer. *Int. J. Colorect. Dis.*, **6**, 115-120.

LINDMARK G, GERDIN B, PÅLHMAN L AND GLIMELIUS B. (1994). Prognostic predictors in colorectal cancer. *Dis. Colon Rectum*, **37**, 1219-1227.

LIOTTA LA, STETLER-STEVENSON W AND STEEG PS. (1991). Metastasis suppressor genes. *Important Adv. Oncol.*, 85-100.

LOMBARDI D, SACCHI A, D'ANGOSTINO G AND TIBURSI G. (1995). The association of the Nm23-M1 protein and beta-tubulin correlates with cell differentiation. *Exp. Cell Res.*, **217**, 267-271.

MANDAI M, KONISHI I, KOSHIYAMA M, MORI T, ARAO S, TASHIRO H, OKAMURA H, NOMURA H, HIAI H AND FUKUMOTO M. (1994). Expression of metastsis-related nm23-H1 and nm23-H2 genes in ovarian carcinomas: correlation with clinicopathology, EGFR, c-*erb*B-2, and c-*erb*B-3 genes, and sex steroid receptor expression. *Cancer Res.*, **54**, 1825-1830.

MORSON BC AND SOBIN LH. (1976). *Histological Typing of Intestinal Tumours*. International Classification of Tumours No. 15. World Health Organization: Geneva.

MYEROFF LL AND MARKOWITZ SD. (1993). Increased nm23-H1 and nm23-H2 messenger RNA expression and absence of mutations in colon carcinomas of low and high metastatic potential. *J. Natl Cancer Inst.*, **85**, 147-152.

NAKAYAMA H, YASUI K, YOKOZAKI H AND TAHARA E. (1993). Reduced expression of nm23 is associated with metastasis of human gastric carcinomas. *Jpn. J. Cancer Res.*, **84**, 184-190.

OKADA K, URANO T, GOI T, BABA H, YAMAGUCHI A, FURUKAWA K AND SHIKU H. (1994). Isolation of human nm23 genomes and analysis of loss of heterozygosity in primary colorectal carcinomas using a specific genomic probe. *Cancer Res.*, **54**, 3979-3982.

PETO R, PIKE MC, ARMITAGE P, BRESLOW NE, COX DR, HOWARD SV, MANTEL N, MCPHERSON K, PETO J AND SMITH PG. (1977). Design and analysis of randomized clinical trials requiring prolonged observation of each patient II. Analysis and examples. *Br. J. Surg.*, **35**, 1-39.

ROYDS JA, STEPHENSON TJ, REES RC, SHORTHOUSE AJ AND SILCOCKS PB. (1993). Nm23 protein expression in ductal in situ and invasive human breast carcinoma. *J. Natl Cancer Inst.*, **85**, 727-731.

ROYDS JA, CROSS SS, SILCOCKS PB, SCHOLEFIELD JH, REES RC AND STEPHENSON TJ. (1994). Nm23 'anti-metastatic' gene product expression in colorectal carcinoma. *J. Pathol.*, **172**, 261-266.

SASTRE-GARAU X, LACOMBE ML, JOUVE M, VÉRON M AND MAGDALÉNAT H. (1992). Nucleoside disphosphate kinase/nm23 expression in breast cancer: lack of correlation with lymph-node metastasis. *Int. J. Cancer*, **50**, 533-538.

STAHL JA, LEONE A, ROSENGARD AM, PORTER L, KING CR AND STEEG PS. (1991). Identification of a second human nm23 gene, nm23-H2. *Cancer Res.*, **51**, 445-449.

STEEG PS, BEVILACQUA G, KOPPER L, THORGEIRSSON UP, TALMADGE E, LIOTTA LA AND SOBEL ME. (1988). Evidence for a novel gene associated with low tumor metastatic potential. *J. Natl Cancer Inst.*, **80**, 200-204.

STEEG PS, COHN KH AND LEONE A. (1991). Tumor metastasis and nm23: current concepts. *Cancer Cells*, **3**, 257-262.

TANNAPFEL A, KOCKERLING F, KATALINIC A AND WITTEKIND C. (1995). Expression of nm23-H1 predicts lymph node involvement in colorectal carcinoma. *Dis. Colon Rectum*, **38**, 651-654.

TOKUNAGA Y, URANO T, FURUKAWA K, KONDO H, KANEMATSU T AND SHIKU H. (1993). Reduced expression of nm23-H1, but not of nm23-H2, is concordant with the frequency of lymph-node metastasis of human breast cancer. *Int. J. Cancer*, **55**, 66-71.

WANG L, PATEL U, GHOSH L, CHEN H-C AND BANERJEE S. (1993). Mutation in the nm23 gene is associated with metastasis in colorectal cancer. *Cancer Res.*, **53**, 717-720.

WHITELAW JL AND NORTHOVER JMA. (1994). The nm23 gene and colorectal cancer. *Gut*, **35**, 141.

XERRI L, GROB JJ, BATTYANI Z, GOUVERNET J, HASSOUN J AND BONERANDI JJ. (1994). NM23 expression in metastasis of malignant melanoma is a predictive prognostic parameter correlated with survival. *Br. J. Cancer*, **70**, 1224–1228.

YAMAGUCHI A, URANO T, FUSHIDA S, FURUKAWA K, NISHIMURA G, YONEMURA Y, MIYZAKI I, NAGAKAWA G AND SHIKU H. (1993). Inverse association of nm23-H1 expression by colorectal cancer with liver metastasis. *Br. J. Cancer*, **68**, 1020–1024.

ZENG ZS, HSU S, ZHANG ZF, COHEN AM, ENKER WE AND TURNBULL AA. (1994). High level of nm23-H1 gene expression is associated with local colorectal cancer progression not with metastases. *Br. J. Cancer*, **70**, 1025–1030.